# Wearable abdominal acoustic biofeedback for dietary self-regulation among Taiwanese university students: an exploratory randomized pilot study

**DOI:** 10.3389/fnut.2026.1799873

**Published:** 2026-09-25

**Authors:** Chen Han, Sheng-Ming Wang, Chuan-Chia Wang, Jwo-Shiun Sun, Tung-Jing Fang

**Affiliations:** 1 Doctoral Program in Design, College of Design, National Taipei University of Technology, Taipei, Taiwan; 2 Department of Interaction Design, National Taipei University of Technology, Taipei, Taiwan; 3 Department of Electronic Engineering, National Taipei University of Technology, Taipei, Taiwan; 4 Department of Physiology and Biophysics, Graduate Institute of Physiology, College of Medicine, National Defense Medical University, Taipei, Taiwan

**Keywords:** abdominal sounds, cultural context, dietary monitoring, interoceptive awareness, self-regulation, university students, user-centered design, wearable sensors

## Abstract

**Background:**

Manual dietary self-monitoring can impose a burden on university students, limiting sustained engagement with digital nutrition tools. AbSound was developed as a wearable abdominal-acoustic biofeedback system that converts candidate gastrointestinal sound events into uncertainty-aware reflective prompts, rather than validated diagnoses of hunger, satiety, dietary intake, diet quality, or eating speed. This exploratory, randomized pilot study assessed feasibility, acceptability, user experience, preliminary self-reported behavioral signals, and agreement in acoustic event detection among Taiwanese university students.

**Method:**

Sixty first- and second-year university students in Taipei were randomized 1:1 to AbSound plus a companion app or to an active app-based dietary-tracking comparator for 4 weeks. Primary feasibility outcomes included retention, daily wear time, days worn, prompt exposure, prompt acknowledgment, reported burden, and adverse events. Exploratory behavioral outcomes included self-reported late-night eating, perceived eating speed, a brief dietary-quality score, self-regulation of eating behavior, weight, and waist circumference. TAM ratings, Kano feature classifications, brief interviews, and descriptive acceptance-path analyses characterized adoption and design priorities. Acoustic performance was evaluated *post hoc* against selected manually reviewed event segments.

**Results:**

All participants completed follow-up. AbSound users wore the device for 9.2 ± 2.2 h per day on 25.7 ± 2.0 of 28 study days, corresponding to approximately 91.8% of possible study days. Prompt acknowledgment averaged 78% ± 9%. Selected, manually reviewed segments showed approximately 88% agreement in event detection, but this estimate does not establish physiological-state validation. Compared with the active comparator, AbSound users showed larger exploratory reductions in self-reported late-night eating. They reported lower perceived eating-speed scores, higher brief dietary-quality scores, and a marginally greater increase in eating self-regulation. Weight and waist circumference showed no clinically interpretable between-group differences. Acceptance ratings were high; comfort and privacy emerged as must-be design attributes, while feedback agreement and timeliness were performance attributes.

**Conclusion:**

AbSound demonstrated short-term feasibility and acceptability as a reflective abdominal-acoustic biofeedback tool for non-clinical Taiwanese university students. The findings support further user-centered development but do not establish clinical efficacy or validated detection of nutritional or physiological states. Larger preregistered trials require independent acoustic validation, objective or repeated dietary reference measures, longer follow-up, and explicit psychological-safety monitoring.

## 1 Introduction

Digital nutrition tools can support goal setting, self-monitoring, and dietary self-regulation (1–4). The practical limitation is not simply a lack of nutrition information. Many systems still require manual food logging, portion estimation, repeated decision entry, and retrospective recall. This burden is especially salient for university students, whose eating routines are shaped by irregular schedules, academic stress, social dining, late-night studying, and easy access to convenience foods (5, 6).

Wearable dietary-monitoring research has sought to reduce this burden by passively or semi-passively sensing acoustic, motion, image, and physiological signals (7–9). These systems continue to face unresolved problems in ecological validity, privacy, signal-to-noise ratio, behavioral interpretability, and user trust. The present study, therefore, asks a narrower HCI and digital-nutrition question: whether uncertain abdominal-acoustic events can be translated into low-burden reflective prompts that users can accept, ignore, postpone, or reinterpret in daily campus contexts.

The AbSound system was developed as an abdominal-acoustic biofeedback interface, not as an objective dietary-intake sensor. The technology can detect candidate abdominal-sound events and translate them into check-in invitations for meal timing, fullness, and perceived eating pace. The central objective of this study is to design and conduct the preliminary evaluation of an uncertainty-aware reflective interface. The system can prompt the user to pause; however, it does not claim the ability to diagnose hunger, satiety, caloric intake, dietary quality, or objective eating speed. Consequently, the empirical assertions of this manuscript are constrained to feasibility, acceptability, user experience, preliminary self-reported behavioral signals, and *post hoc* event-detection agreement within a non-clinical student population.

### 1.1 Abdominal borborygmi as novel dietary monitoring signals

Conventional dietary monitoring instruments, such as calorie calculators, food diaries, and photo-based applications, primarily monitor external behaviors, including food type, timing, and portion size (2, 3). Abdominal sounds provide a complementary interface for internal signals because gastrointestinal motility and digestion can produce measurable acoustic events. However, it should be noted that these events are variable, context-dependent, and vulnerable to movement artifacts, environmental noise, sensor placement, and individual physiological differences (10, 11).

The behavioral rationale underpinning this phenomenon is rooted in the principles of reflective self-regulation and interoceptive awareness. It does not assert a direct correlation between abdominal sounds and the physiological sensations of hunger or satiety (12–14). A prompt may help a user pause to examine their bodily sensations before eating. However, an increased focus on internal signals could also lead to heightened vigilance, confusion, guilt, or compulsive checking among vulnerable users (15–17). This safety boundary is pivotal to the design interpretation.

Recent research in abdominal sound studies has indicated the potential of machine-learning methods to identify significant bowel sound events under controlled or semi-controlled conditions (10, 11). The issue under discussion in this paper is distinct: namely, whether such event candidates can be integrated into a user-centered dietary self-reflection workflow. Consequently, the prompts issued by AbSound were formulated as reflective check-ins rather than as deterministic instructions.

### 1.2 Study context and theoretical framework

The context of Taiwanese university students is pertinent to the study of low-burden dietary self-monitoring. The campus routines of these students frequently entail late meals, convenience foods, variable sleep schedules, and familiarity with mobile technology (5, 6). The cultural context is utilized to provide a framework for interpreting recruitment, privacy expectations, and design fit (18, 19).

The conceptual framework under consideration integrates three components. Firstly, user-centered design and Kano analysis are employed to identify adoption requirements, including comfort, privacy, discretion, prompt burden, and perceived feedback agreement (20–22). Secondly, the Technology Acceptance Model (TAM) provides an HCI adoption lens through perceived ease of use, perceived usefulness, trust, and behavioral intention (23–25). Thirdly, the theoretical underpinnings of self-regulation and interoceptive awareness postulate mechanisms through which prompts may foster reflective eating decisions (12, 14, 26). These frameworks are used to organize findings on feasibility and acceptance.

Cross-regional transfer, clinical translation, and preventive-care implementation were not empirically tested in this dataset. It is therefore recommended that future research should focus on the following questions: cultural adaptation, privacy norms, peer influence, long-term engagement, and safety in vulnerable users.

To maintain conceptual focus, the present manuscript restricts the empirical claim to a non-clinical student pilot. Prior AbSound development work in other populations is not used as evidence for the dietary outcomes reported here.

Study objectives: This exploratory pilot addressed five feasibility- and development-oriented objectives:

1. Estimate short-term feasibility, including retention, daily wear time, days worn, prompt exposure, prompt acknowledgment, reported burden, and adverse events.
2. Characterize acceptability and user experience using TAM ratings, Kano feature classifications, qualitative feedback, comfort, privacy, and willingness to continue.
3. Compare preliminary changes in self-reported late-night eating, perceived eating speed, brief dietary-quality score, and eating self-regulation between AbSound and an active app-based dietary-tracking comparator.
4. Estimate preliminary acoustic event-detection agreement and describe its ecological limitations.
5. Identify acoustic-validation, psychometric, trial-design, and ethical requirements that must be addressed before efficacy testing or clinical translation.

The exploratory acceptance hypotheses were applied exclusively to the AbSound group and used to organize descriptive HCI findings. Future efficacy testing would require an adequately powered, preregistered trial with independent acoustic validation, objective or repeated dietary reference measures, and explicit psychological-safety monitoring.

## 2 Method

### 2.1 Study design and participants

A four-week, two-arm exploratory randomized pilot trial was conducted among first- and second-year university students in Taipei, Taiwan (*N* = 60; 60% female; mean age 19.5 ± 0.6 years). Participants were recruited through campus flyers and screened for eligibility, including the absence of diagnosed dietary or gastrointestinal disorders and no use of appetite-affecting medications. Participants were assigned in a 1:1 ratio to AbSound plus companion app or to an active app-based dietary-tracking comparator. The primary estimands in this report are feasibility and acceptability measures rather than efficacy estimands. The sample was selected to estimate feasibility, acceptability, user-experience signals, and preliminary outcome variability, consistent with pilot-study logic (27, 28). All randomized participants completed follow-up; therefore, randomized, complete-case, and available-case analyses included the same 60 participants.

### 2.2 Randomization and registration

Participants were assigned in a 1:1 ratio after eligibility confirmation. The available pilot documentation does not permit a complete retrospective reconstruction of how the allocation sequence was generated, who generated it, how assignment was implemented, or whether allocation concealment was used before group assignment. Allocation concealment should therefore be treated as undocumented in the interpretation of this randomized pilot. No prospective trial-registration record was available. The participant-flow presentation and reporting framework were assessed with reference to CONSORT guidance for parallel-group randomized trials (50, 51). These limitations are stated explicitly to avoid overstating the strength of causal inference (Figure 1).

**Figure 1:**
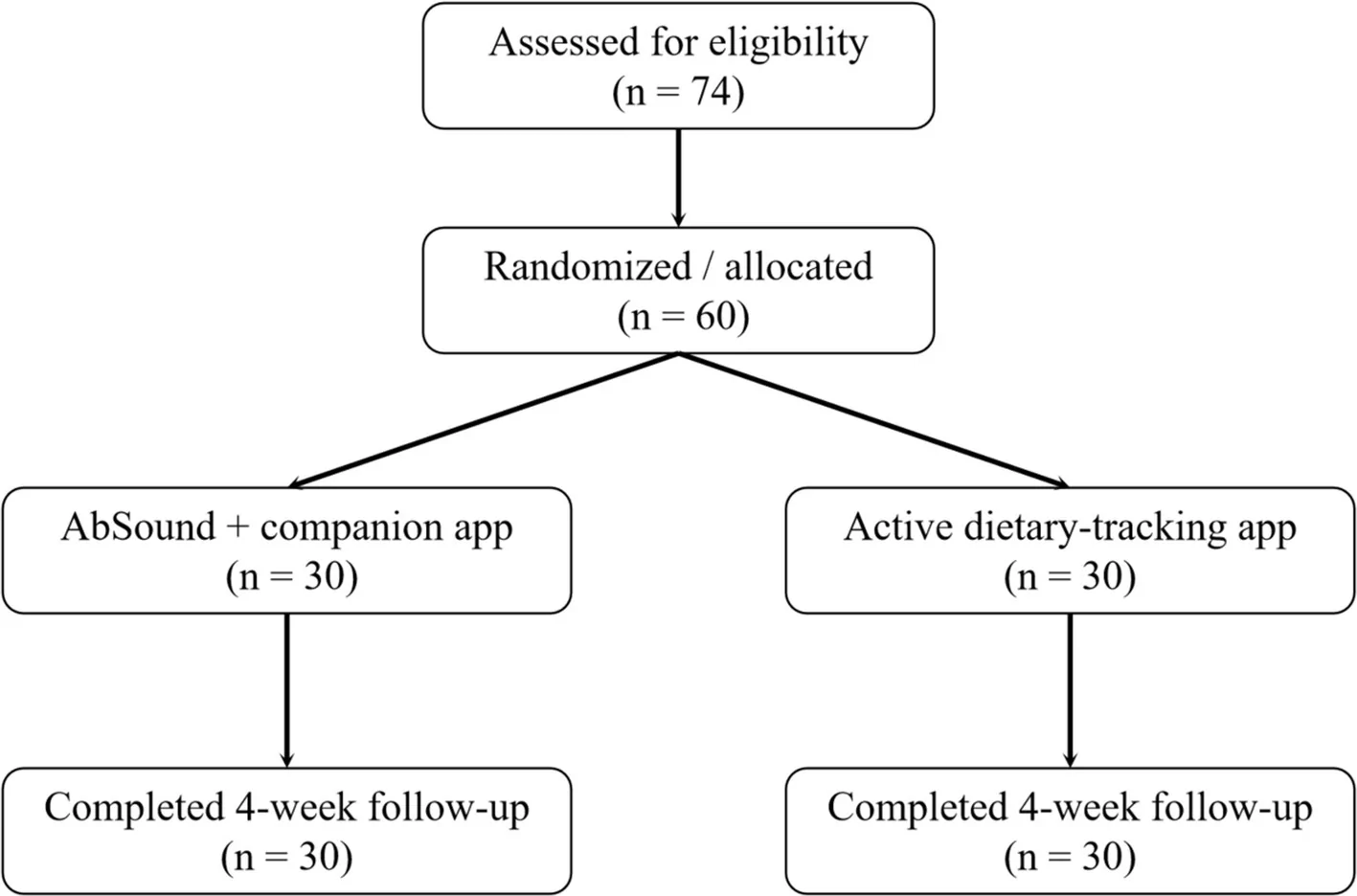
Four-week randomized trial design and participant flow. A total of 74 students were assessed for eligibility, and 60 were allocated in a 1:1 ratio to either AbSound plus the companion app or an active dietary-tracking app. All 60 participants completed the follow-up.

### 2.3 Ethics and consent

The study protocol was approved by the university’s Institutional Review Board (IRB No. KY2021-609). In accordance with the stipulated Ethics Statement, written informed consent was not a prerequisite under the prevailing local legislation and institutional requirements. All participants provided written informed consent before participation. Prior to participation, students were provided with study information and informed that AbSound recorded contact abdominal acoustics as opposed to speech, that feedback categories were exploratory prompts as opposed to medical or nutritional diagnoses, and that they had the option to pause alerts, remove the device, decline prompt responses, or withdraw at any time. The stored research data were analyzed in de-identified form. The necessity for these safeguards arises from the potential of self-monitoring tools to modify perceived responsibility, privacy expectations, and reliance on automated feedback (29–32).

The baseline characteristics for both groups are outlined in Table 1. The two groups, i.e., the wearable group and the control group, were comparable in terms of age, BMI, waist circumference, baseline diet-quality score, and self-regulation of eating. The demographic profile of the participants is as follows: 60% were female, approximately 92% resided in on-campus housing, and approximately 29% had prior experience with wearable devices such as fitness trackers.

**Table 1:** Participant baseline characteristics (mean ± SD or %).

Characteristic	Wearable group (*n* = 30)	Control group (*n* = 30)	Overall (*N* = 60)
Age (years)	19.4 ± 0.5	19.6 ± 0.6	19.5 ± 0.6
Female (%)	67%	53%	60%
BMI (kg/m^2^)	21.9 ± 1.6	21.2 ± 2.0	21.6 ± 1.8
Overweight (%) (BMI ≥ 24)	7%	7%	7%
Waist circumference (cm)	80.3 ± 7.1	78.9 ± 6.5	79.6 ± 6.8
Diet quality score (0–100)	59.6 ± 7.8	61.7 ± 9.9	60.7 ± 8.9
Self-regulation of eating (SREB)	2.94 ± 0.47	2.83 ± 0.44	2.89 ± 0.46
Lives on campus (%)	90%	93%	92%
Prior wearable use (%)	30%	27%	29%

Self-Regulation of Eating Behavior scale, 1–5 (higher = better self-regulation).

### 2.4 Intervention

*AbSound Wearable System:* The intervention group received the AbSound system, comprising: (1) a lightweight abdominal acoustic sensor worn via an elastic waistband, and (2) a companion smartphone application. The sensor module incorporates a high-sensitivity contact microphone (digital stethoscope) positioned on the abdomen to capture internal sounds. We recommend two optimal placement locations: the left upper quadrant (LUQ), below the ribs (where gastric “growling” is prominent), or the right lower quadrant (RLQ), near the ileocecal valve (intestinal sound source), based on comfort. The device connects to the smartphone app via low-energy Bluetooth (BLE 5.0).

Analog audio signals were filtered and digitized internally (sampling rate: 1,000 Hz; 50–800 Hz band-pass filter). The raw acoustic data were streamed to the application and processed using a lightweight convolutional neural network to classify 10-s segments into exploratory prompt categories: candidate pre-meal abdominal activity, post-meal digestive activity, or non-target/noise. It is important to note that these categories do not equate to clinically validated states of hunger, satiety, dietary intake, diet quality, or eating speed. The approximate 88% estimate reported hereinafter pertains to *post hoc* agreement between model-generated event labels and selected audio/event segments that underwent manual review.

When the application identified candidate abdominal activity, it delivered reflective prompts rather than deterministic instructions. For instance, a pre-meal check-in could state, “Your stomach is active; check whether you feel hungry before deciding what to do next.” A post-prandial check-in could state, “Your abdomen shows increased post-prandial activity; consider pausing and checking fullness.” Users can acknowledge, postpone, silence, or ignore alerts. The wording was meticulously crafted to promote self-regulation while precluding unsubstantiated claims that a single acoustic event can serve as an indicator of hunger, satiation, overeating, or objectively rapid eating.

The mobile application also offered data logging and dashboard functions, enabling users to access a timeline of candidate prompt events and weekly summaries, encompassing late-evening prompt frequency and alert acknowledgment. The data were stored locally on the mobile device, with an optional encrypted backup used solely for research analysis. The application required no continuous manual food entry beyond optional meal notes and prompt responses, which was intended to reduce user burden relative to conventional food diaries.

See Table 2 for detailed specifications of the wearable devices.

**Table 2:** AbSound wearable device specifications.

Item	Specification
Sensor type	Contact microphone (piezoelectric)
Sampling rate	1,000 Hz
Frequency band-pass	50–800 Hz (bowel sound range)
Module dimensions	~5 × 5 × 1.5 cm
Module weight	~50 g
Wearing method	Sensor; LUQ or RLQ placement
Battery life	~10 h continuous use
Connectivity	Bluetooth Low Energy (BLE 5.0)
On-device processing	Real-time filtering; frame segmentation
Mobile platform	Android (and iOS compatible)
Data handling	On-phone analysis; derived features stored; optional encrypted backup

The comparator group used a widely available dietary-tracking application and was instructed to record dietary intake and weight as usual while receiving the application’s standard automated feedback. At study entry, both groups received generic healthy-eating advice, including guidance on regular mealtimes, reducing late-night snacks, and moderating eating pace. The primary contrast was therefore AbSound plus low-burden abdominal-acoustic reflective prompts versus active app-based dietary self-monitoring. Behavioral contrasts should be interpreted as preliminary signals relative to this active comparator, not relative to no intervention.

### 2.5 User-centered design and training

Participants in the AbSound group underwent a 30-min training session covering device placement, app use, troubleshooting, comfort, privacy safeguards, and the significance and limitations of prompts. The design process incorporated brief co-design discussions with students prior to and during the pilot phase, adhering to principles of usability and person-based design (20, 22, 33). Participants provided commentary on the following aspects: the wording of alerts, the modalities of notifications, privacy concerns, the comfort of the sensors, the ability to conceal them under clothing, their use in a classroom setting, and the interpretability of dashboards. Iterative refinements included the following:

- Lower notification volume.
- Vibration-only alert options.
- Simplified explanatory text.
- Clearer wording that prompts were reflective rather than diagnostic.

Participants were asked to wear the device while awake when feasible, with a recommended target of at least 8 h daily covering pre-meal, meal, and post-meal periods. The device could be removed during exercise, in cases of discomfort, or in situations where privacy was deemed sensitive. It could also be removed in any situation where wearing it was considered inappropriate. The application monitored usage duration via Bluetooth connectivity and device-status indicators. Study personnel contacted participants weekly to troubleshoot any issues and monitor the study’s burden.

### 2.6 Measurement metrics

Quantitative and qualitative data were collected at baseline and 4 weeks.

### 2.7 Dietary behavior outcomes

The exploratory behavioral outcomes were measured at baseline and 4 weeks.

### 2.8 Nighttime eating frequency

Participants self-reported the weekly number of nighttime eating episodes after 10 p.m.

### 2.9 Perceived eating speed

Participants rated perceived eating speed on a single 5-point Likert item (1 = very slow, 5 = very fast). Lower follow-up scores indicate slower perceived eating. This item was not used to infer binge eating, compulsive control, or objectively measured meal pace.

### 2.10 Dietary quality

A brief dietary-quality score (0–100) summarized fruit and vegetable frequency, sugar-sweetened beverage intake, and fried-food consumption. Higher scores indicate better self-reported dietary quality.

### 2.11 Self-regulation of eating

The Self-Regulation of Eating Behavior Scale (SREB) yielded a composite score from 1 to 5, reflecting perceived ability to regulate eating habits such as planning regular meals and resisting unplanned snacking. Higher scores indicate stronger perceived self-regulation.

We concurrently tracked weight and waist circumference, though no significant changes were anticipated during the 4-week trial phase. Weight was measured using calibrated scales at baseline and follow-up (light clothing, barefoot).

### 2.12 Device usage metrics

For the intervention group, the app recorded objective usage data, including daily wear duration, days worn, prompt exposure, prompt acknowledgment, and candidate event counts.

- Daily wear duration (hours per day).
- Wear compliance days: Number of days within the 28-day period when the device was actually worn for part of the time.
- Number of candidate pre-meal and post-meal check-in prompts sent.
- Alert acknowledgment rate: Proportion of users who tapped “Received” (acknowledged) versus ignored alerts.
- Algorithm performance: *Post hoc* manual comparison of app annotations with selected audio/event segments was used to estimate preliminary event-detection agreement. This analysis should not be interpreted as validation of physiological hunger, satiety, dietary intake, diet quality, or the detection of eating speed.

See the Results section for a summary of relevant metrics (refer to Table 3; Figure 2).

- Technology acceptance and user feedback: We administered a post-intervention questionnaire to the intervention group covering core constructs of the Technology Acceptance Model (TAM):
- Perceived Ease of Use (PEOU): 5 indicators (e.g., “The system is easy to learn,” “Using the system fits naturally into my daily routine”).
- Perceived Usefulness (PU): 5 items (e.g., “The system helps me self-regulate my diet,” “System feedback prompts me to make healthier choices”).
- Trust: 4 items (e.g., “I trust the accuracy of the system’s detection results,” “System explanations enhance my confidence in the feedback”).
- Behavioral Intention: 3 items (e.g., “I will continue using the system if it remains available,” “I would recommend it to friends”).

**Table 3:** Device usage and engagement metrics (intervention group, *N* = 30).

Metric	Value (mean ± SD)
Daily wear time (hours/day)	9.2 ± 2.2 h
Days worn (out of 28)	25.7 ± 2.0 days (91.8%)
Pre-meal abdominal-activity alerts received (4 weeks)	21.5 ± 6.1 alerts
Post-meal activity/check-in alerts received	17.7 ± 6.0 alerts
Alerts acknowledged (%)	78% ± 9%
Eating-related/prompt-relevant events flagged (4 weeks)	79 ± 8 episodes
Preliminary event-detection agreement	Approx. 88% agreement on selected manually reviewed event segments; not physiological-state validation

**Figure 2:**
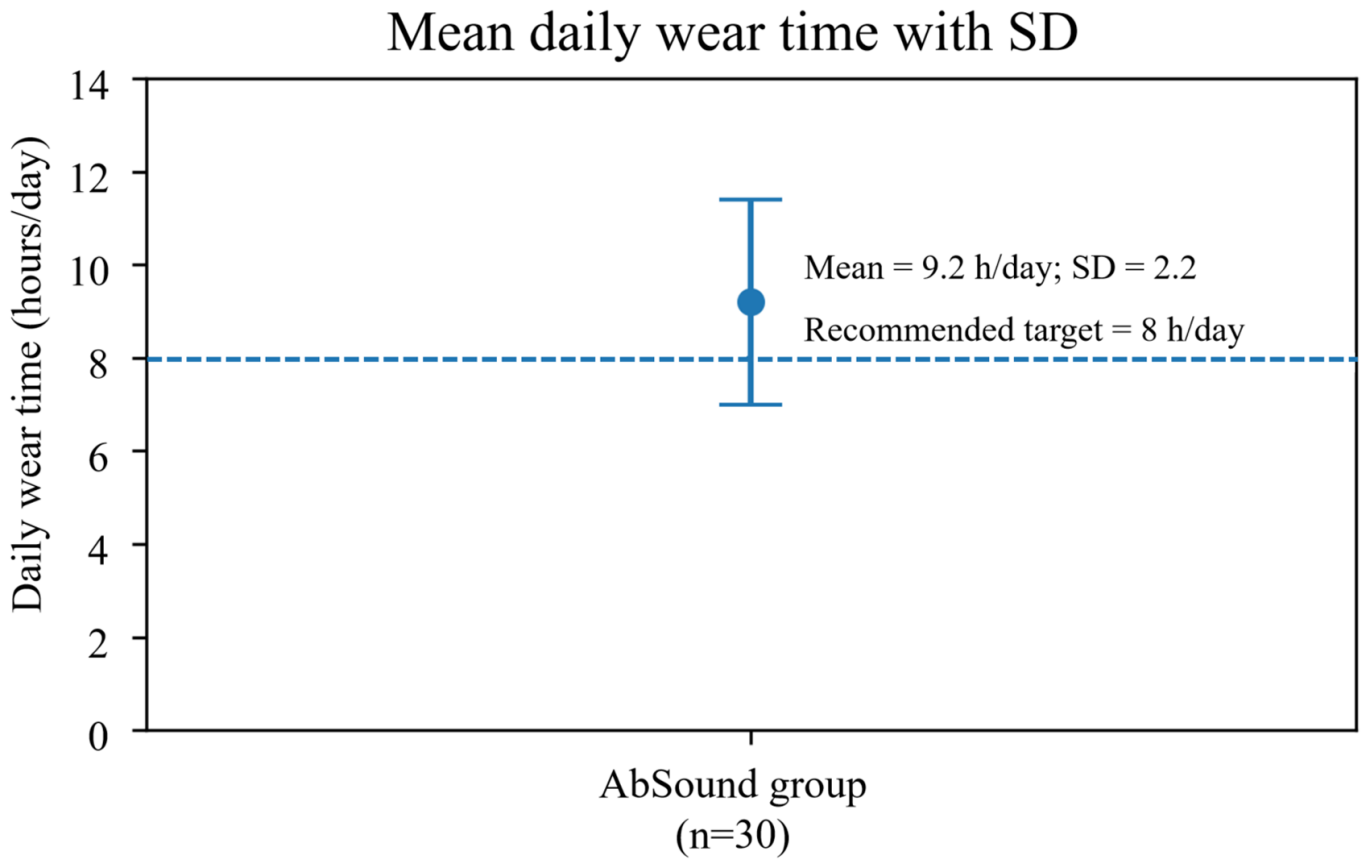
Mean daily wear time in the AbSound group. The point indicates the mean, the error bar indicates the SD, and the dashed line indicates the recommended target of 8 hours per day. The figure is descriptive and supports the feasibility endpoint reported in Table 3.

Each item was rated on a 5-point Likert scale (1 = strongly disagree, 5 = strongly agree). The items under discussion were adapted from existing TAM instruments and tailored to the AbSound context (23, 25). Because the measures were adapted and the intervention group was small (*n* = 30), construct scores were reported descriptively. It is recommended that item-level internal consistency and measurement validity should be reported in larger confirmatory samples rather than being inferred from this pilot (34).

Two contextual user characteristics were also measured. The assessment of interoceptive awareness related to hunger and fullness was conducted using a set of six adapted items from the Multidimensional Assessment of Interoceptive Awareness (MAIA), encompassing the recognition of early hunger and the acknowledgment of comfortable fullness (14). The evaluation of eHealth literacy was undertaken using eight items adapted from the eHealth Literacy Scale, which assesses confidence in interpreting health app data and in safeguarding privacy when using health technology (35). In this pilot study, both composites were utilized as exploratory contextual variables, rather than as fully validated moderators.

At the conclusion of the study, participants in the AbSound group completed a Kano-model questionnaire. This questionnaire was used to assess various factors, including comfort, privacy, perceived detection agreement, prompt timeliness, feedback interpretability, interface usability, gamification, and concealment. In this study, participants were presented with paired questions concerning both functional and dysfunctional attributes. The categorization of features was approached as a means of demonstrating design priorities rather than as evidence of behavioral efficacy or physiological validity.

Qualitative feedback was collected through brief semi-structured interviews (15–20 min) with each AbSound participant. Interviews addressed user experience, habit formation, perceived strengths and weaknesses, false or poorly timed alerts, discomfort, privacy, classroom use, prompt burden, and suggestions for improvement. Audio-recorded interviews were analyzed using a rapid thematic approach (36–38).

### 2.13 Exploratory descriptive acceptance-path analysis

TAM-related variables were available only in the AbSound group (*n* = 30), so a confirmatory structural equation model was not fitted. We inspected descriptive standardized associations among composite scores to determine whether the observed pattern was broadly consistent with TAM-oriented adoption hypotheses. These analyses were hypothesis-generating only; latent-variable interpretation, model-fit claims, and causal interpretation were intentionally avoided.

The exploratory paths inspected included perceived ease of use to perceived usefulness, trust to perceived usefulness, perceived usefulness to engagement, perceived usefulness to change in SREB, interoceptive awareness to perceived usefulness or engagement, and eHealth literacy to engagement.

The small sample, post-intervention-only TAM data, adapted measures, and multiple inspected paths make these analyses unstable and susceptible to overfitting. Future studies should test simplified, preregistered acceptance models in adequately powered samples and include independent validation cohorts.

### 2.14 Data analysis

The primary analytic focus was system feasibility, including retention, daily wear time, days worn, prompt counts, prompt acknowledgment, adverse events, and reported burden. Secondary behavioral outcomes were exploratory. Between-group changes were compared using *t*-tests or Mann–Whitney U tests when distributional assumptions were not plausible; within-group pre-post changes were examined descriptively. Categorical variables were compared using chi-square tests. *p*-values were not adjusted for multiple comparisons and should be interpreted as descriptive. Table 4 reports baseline and follow-up means with SDs, unadjusted difference-in-change estimates, approximate 95% confidence intervals where estimable, unadjusted *p*-values, and interpretive boundaries. Standardized effect-size estimates are not emphasized because the pilot was small and not designed for stable effect-size estimation.

**Table 4:** Exploratory behavioral outcomes in the AbSound and active-comparator groups before and after the four-week pilot.

Outcome	AbSound baseline → follow-up	Active comparator baseline → follow-up	Descriptive difference in mean change	Interpretive boundary
Late-night snacks (episodes/week)	3.29 ± 0.49 → 2.07 ± 0.47; Mean change −1.22	2.98 ± 0.48 → 2.82 ± 0.81; Mean change −0.16	−1.06 episodes/week	Self-reported; exploratory signal relative to the active comparator
Perceived eating speed (1 = very slow, 5 = very fast)	3.48 ± 0.87 → 2.97 ± 0.53; Mean change −0.51	3.27 ± 0.86 → 3.22 ± 1.39; Mean change −0.05	−0.46 points	Self-reported; lower scores indicate slower perceived eating, not observed meal pace.
Brief dietary-quality score (0–100)	59.6 ± 7.8 → 67.8 ± 8.5; Mean change +8.2	61.7 ± 9.9 → 64.1 ± 5.1; Mean change +2.4	+5.8 points	Brief self-report; requires validation against a dietary reference measure
Self-regulation of eating (SREB, 1–5)	2.94 ± 0.47 → 3.35 ± 0.68; Mean change +0.41	2.83 ± 0.44 → 2.99 ± 0.94; Mean change +0.16	+0.25 points	Self-reported; directional and hypothesis-generating, not a confirmed between-group effect
Weight (kg)	60.2 ± 1.2 → 60.1 ± 1.4; Mean change −0.1	59.5 ± 1.6 → 60.0 ± 1.2; Mean change +0.5	−0.6 kg	Descriptive only; no weight-management efficacy conclusion
Waist circumference (cm)	80.3 ± 7.1 → 79.9 ± 9.5; Mean change −0.4	78.9 ± 6.5 → 79.3 ± 6.7; Mean change +0.4	−0.8 cm	Clinically uninterpretable over 4 weeks; reported for completeness

Reporting framework and feasibility estimates: The primary estimands were feasibility and acceptability measures, including retention, days worn, daily wear time, prompt exposure, prompt acknowledgment, self-reported burden, and adverse events (27, 39, 40). Exploratory behavioral contrasts were summarized as unadjusted difference-in-change estimates for signal detection and future sample-size planning, rather than as definitive treatment effects (28, 41).

For qualitative data, a rapid thematic analysis was employed to contextualize the quantitative findings, with attention to comfort, privacy, prompt clarity, perceived burden, perceived usefulness, false or poorly timed prompts, and perceived influence on eating routines. These qualitative findings were utilized to inform interpretation and future design priorities, rather than to establish efficacy (36, 37).

## 3 Results

### 3.1 Device compliance and usage engagement

Short-term feasibility was high. Participants in the AbSound group wore the device for 9.2 ± 2.2 h per day (median: 9.4 h) on 25.7 ± 2.0 of the 28 study days, corresponding to 91.8% of the possible study days (Table 3; Figure 2). All 30 participants in each group completed the four-week follow-up. These findings support short-term feasibility and tolerability.

During the four-week study period, AbSound participants received 21.5 ± 6.1 candidate pre-meal abdominal-activity prompts and 17.7 ± 6.0 post-meal activity or eating-rhythm check-in prompts. Of these prompts, 78% ± 9% were acknowledged via app click or open response. Prompt acknowledgment should not be interpreted as agreement with the physiological meaning of each alert.

The system flagged an average of 79 ± 8 eating-related or prompt-relevant events per participant over 4 weeks. A *post hoc* comparison of selected audio/event segments reviewed manually indicated approximately 88% agreement in event detection. This estimate does not constitute physiological-state validation because class-specific errors, movement artifacts, environmental noise, missingness, and independent reference standards were not fully characterized. No serious device-related adverse events were reported. Several participants reported mild skin irritation in the sensor area during hot weather or sweating. Users also noted that false or missed alerts occurred occasionally in noisy restaurants or during movement. These observations identify comfort, climate, motion artifacts, environmental noise, and alert burden as design constraints for later prototypes.

### 3.2 Behavioral outcomes

The AbSound group showed larger short-term changes than the active comparator across several exploratory, predominantly self-reported eating-behavior measures (Table 4; Figures 3–5). These findings are preliminary behavioral signals; they should not be read as definitive efficacy evidence.

- Night-time eating: Self-reported weekly late-night eating episodes decreased from 3.29 to 2.07 in the AbSound group (change −1.22 episodes) and from 2.98 to 2.82 in the active-comparator group (change −0.16 episodes). The unadjusted difference in change was −1.06 episodes/week (approximate 95% CI −1.86 to −0.26; exploratory *p* = 0.010).

**Figure 3:**
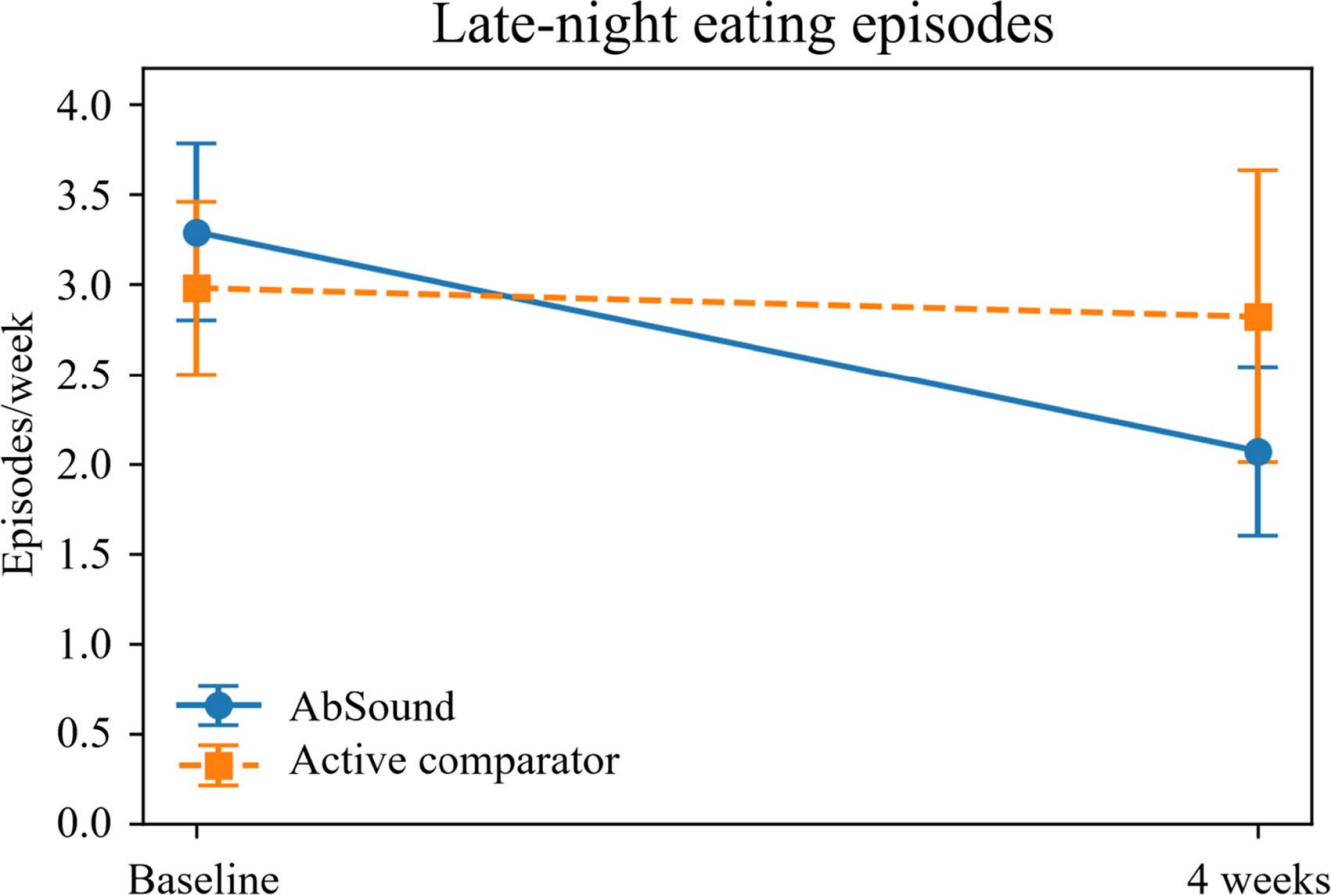
Self-reported weekly late-night eating frequency before and after the pilot in the AbSound and active-comparator groups. Points show means, and error bars show SDs; the figure is descriptive and exploratory.

**Figure 4:**
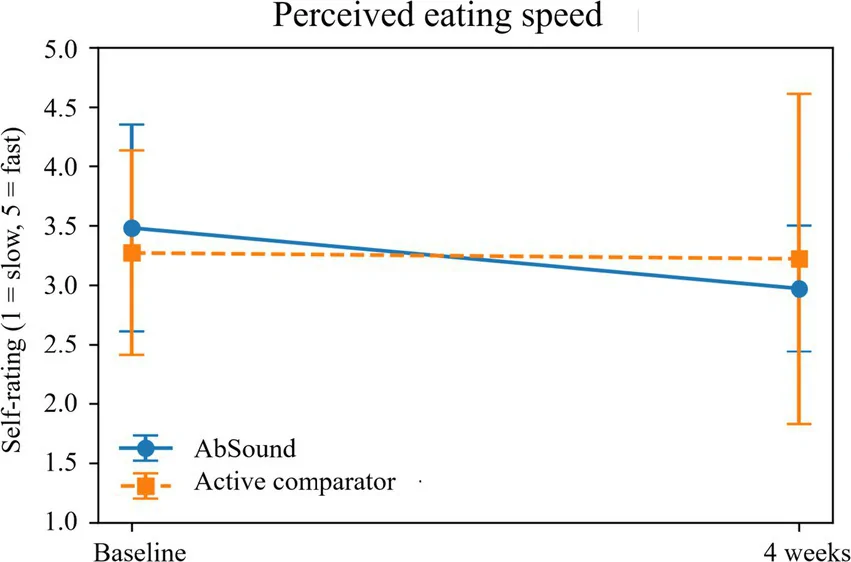
Pre- and post-intervention comparison of perceived eating-speed scores (1 = very slow, 5 = very fast). Points show means, error bars show SDs, and lower scores indicate slower perceived eating. The outcome was not validated against observed meal duration, bite rate, or externally coded eating pace.

**Figure 5:**
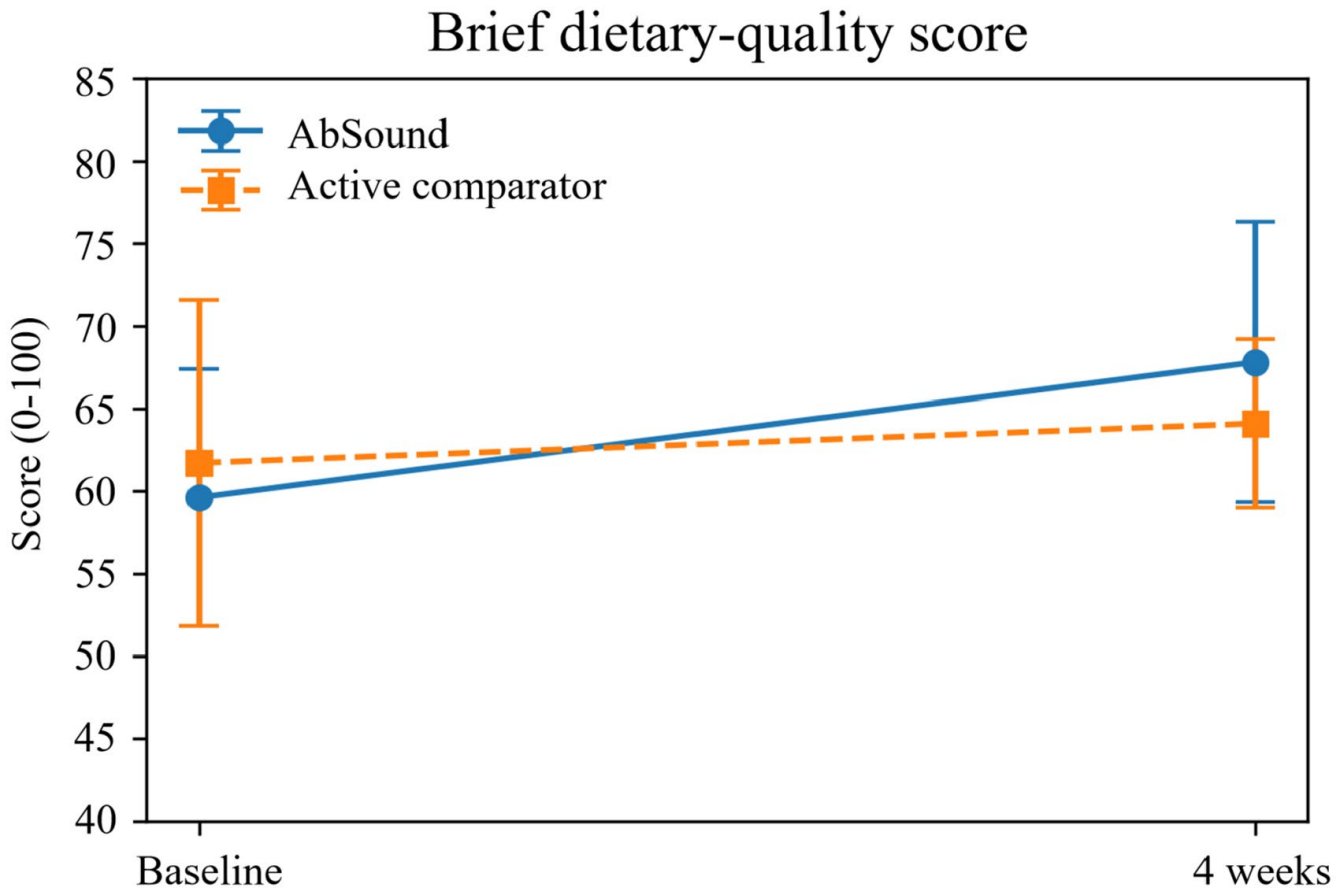
Brief dietary-quality scores before and after the pilot. Points show means, and error bars show SDs. Higher scores indicate better self-reported diet quality on this brief measure.

The mean perceived eating-speed scores decreased from 3.48 to 2.97 in the AbSound group (change −0.51) and from 3.27 to 3.22 in the active-comparator group (change −0.05). As lower scores indicate a slower perceived pace of eating, the observed difference in change (−0.46 points; approximate 95% confidence interval: −0.87 to −0.05; exploratory *p* = 0.030) should be interpreted as a self-reported slowing signal rather than an observed slowing of the meal pace.

- Dietary quality: The brief dietary-quality score increased from 59.6 to 67.8 in the AbSound group (change +8.2) and from 61.7 to 64.1 in the active-comparator group (change +2.4). The unadjusted difference in change was +5.8 points (approximate 95% CI 0.27 to 11.33; exploratory *p* = 0.040). Because the measure was concise and self-reported, validation using repeated dietary recalls, food records, or other reference measures remains necessary.
- Self-Regulated Eating Behavior (SREB): SREB increased from 2.94 to 3.35 in the AbSound group (change +0.41) and from 2.83 to 2.99 in the active-comparator group (change +0.16). The unadjusted difference in change was +0.25 points (approximate 95% CI −0.03 to 0.53; exploratory *p* = 0.080), which should be described as marginal and hypothesis-generating rather than as a clear between-group effect.
- Weight: Mean weight remained stable in the AbSound group and increased slightly in the active-comparator group. The unadjusted difference in change was −0.6 kg (approximate 95% CI −1.42 to 0.22; *p* = 0.150). Given the short duration, small sample, and predominantly normal-BMI cohort, this result should not be interpreted as weight-management efficacy.
- Waist circumference: No clinically interpretable between-group difference was observed; both groups changed by less than 1 cm over 4 weeks. This outcome is reported only for completeness.

Open-ended responses indicated that 18 of 30 AbSound participants (60%) reported at least one perceived behavior change, such as eating breakfast more regularly or reducing late-night consumption of instant noodles, compared with 10 of 30 active-comparator participants (33%). These reports are consistent with the quantitative pattern but remain vulnerable to expectancy, novelty, recall, and social-desirability effects.

Statistical labels are used to denote exploratory complete-case group-by-time comparisons. The behavioral findings suggest that abdominal-acoustic biofeedback may facilitate short-term reflection on eating patterns in a subset of students. Nevertheless, the study does not establish a causal mechanism, objective dietary improvement, or validated physiological interpretation.

In future trials, baseline eating patterns, interoceptive awareness, motivation, and alert burden should be examined as potential moderators using prespecified participant-level criteria.

### 3.3 Technology acceptance model survey results

Post-intervention TAM questionnaires (intervention group, *n* = 30) demonstrated high short-term participant ratings for AbSound. These data are interpreted as evidence of adoption and user experience, consistent with TAM and mobile app quality frameworks, rather than as evidence of acoustic or behavioral efficacy (23, 25, 42, 43).

- Average perceived ease of use (PEOU) was 4.3 ± 0.5 out of 5. The majority of students reported that the system was straightforward to learn and to incorporate into their daily routines, with 93% of respondents agreeing that operating the app required minimal effort.
- Average perceived usefulness (PU) was 4.5 ± 0.4. Students reported that the prompts increased awareness of eating-related cues and helped them reflect on healthier choices. It is important to note that these ratings are indicative of perceived usefulness rather than being based on independently validated physiological accuracy or proven behavioral efficacy.
- Mean trust was 4.2 ± 0.6. Participants frequently reported that notifications generally corresponded to their own sensations; however, it is important to note that subjective agreement should not be equated with technical or physiological validation.
- Behavioral intention averaged 4.6 ± 0.5. Most participants (90%) expressed their intention to continue using AbSound if it becomes available, and 83% would recommend the system to peers. These values indicate intention after a brief pilot phase and should not be interpreted as evidence of long-term adherence.

The TAM constructs showed exploratory correlations in the expected direction: PEOU was positively correlated with PU, and both were positively associated with behavioral intention. Trust showed the strongest association, consistent with the hypothesis that subjective credibility may support adoption. These findings suggest high short-term acceptance, but they do not establish long-term adherence or behavioral efficacy.

### 3.4 Kano analysis

Participants classified system features using the Kano questionnaire (Figure 6). Comfort and privacy were most often classified as must-be attributes, indicating that discomfort or perceived intrusion could prevent adoption. Feedback agreement and timeliness were mainly classified as one-dimensional attributes, suggesting that better performance may increase satisfaction. Gamification elicited more attractive or indifferent responses, suggesting that it is optional rather than central to feasibility. These findings support design prioritization, but should be interpreted cautiously given the small HCI sample (21, 42, 44).

**Figure 6:**
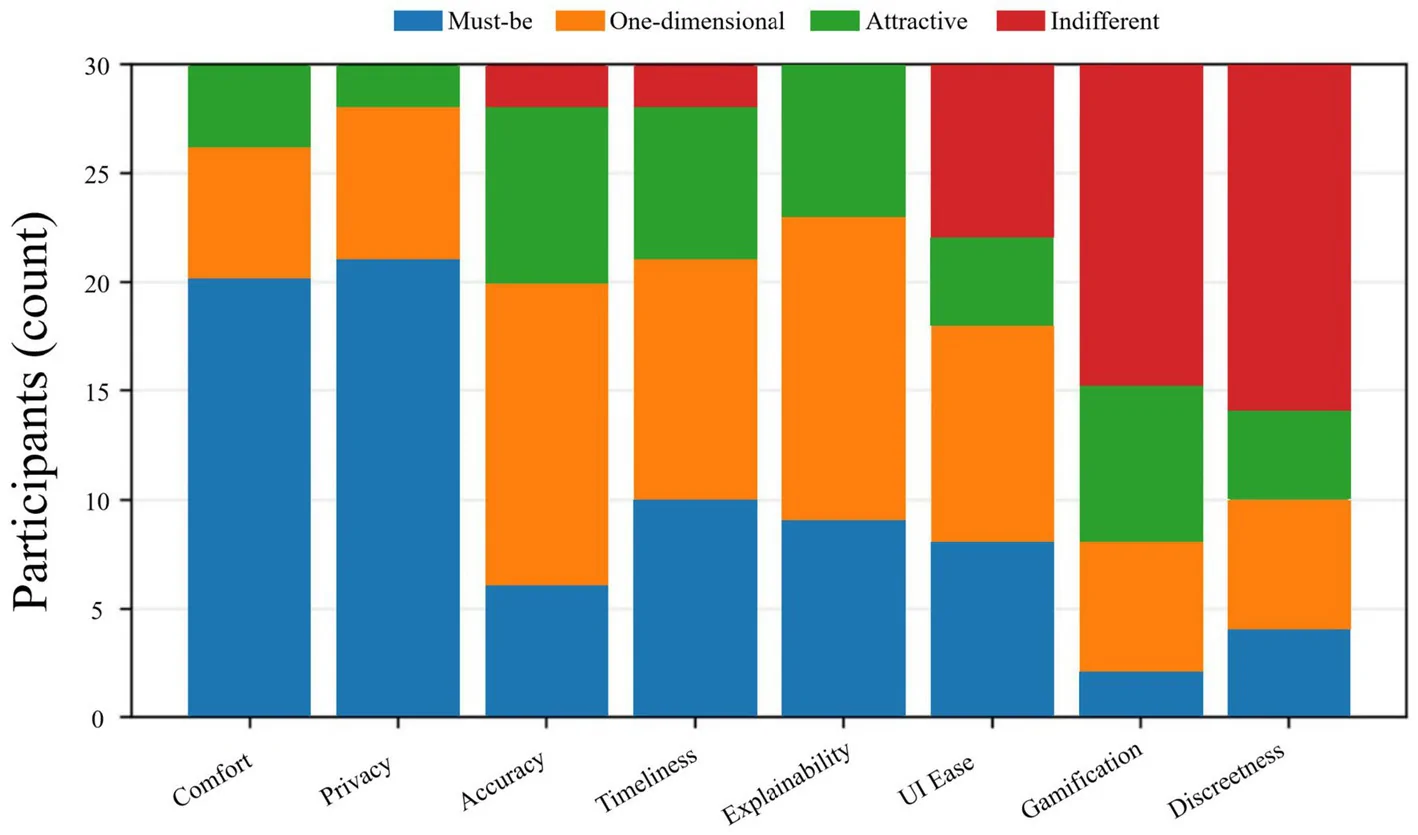
Kano model classification of key system functions in the AbSound group (*n* = 30). Comfort and privacy were predominantly must-be attributes; perceived feedback agreement and timeliness were mostly one-dimensional; gamification and concealment showed more heterogeneous responses.

No feature was predominantly categorized as Reverse. This finding suggests that the included features were not perceived as harmful or undesirable by the majority of users over the 4 weeks. Nevertheless, it does not preclude the possibility of a longer-term burden or subgroup-specific concerns.

### 3.5 Exploratory contextual variables and individual differences

Baseline interoceptive awareness and eHealth literacy were examined only as exploratory contextual variables. Participants with lower baseline interoceptive awareness tended to rate the system as more useful and showed directionally larger self-reported changes in SREB and late-night eating. Students with higher eHealth literacy appeared to integrate AbSound more readily into daily routines. These patterns are underpowered, may be confounded by baseline motivation, and should be tested in preregistered studies using validated moderator measures.

### 3.6 Exploratory descriptive acceptance-path results

Descriptive acceptance-path analyses were conducted within the intervention group only (*n* = 30). Because this was not a confirmatory structural equation model, no model-fit claims are made. The following standardized associations are reported only as pilot-pattern descriptors requiring replication in larger, preregistered samples.

- PEOU → PU: Perceived ease of use was positively associated with perceived usefulness (*β* = 0.51, *p* = 0.008). This descriptive result is consistent with TAM but requires replication in a larger sample.
- Trust → PU: Trust was positively associated with perceived usefulness (*β* = 0.34, *p* = 0.031). Qualitative comments suggested that users relied more on feedback when alerts felt subjectively plausible. This is an acceptance finding, not a validation of algorithmic accuracy.
- PU, engagement, and change in SREB: Perceived usefulness was associated with engagement (*β* = 0.38, *p* = 0.014) and change in SREB (*β* = 0.45, *p* = 0.006). These paths should be read as hypothesis-generating associations, not evidence that perceived usefulness caused behavioral change.
- Interoceptive awareness: The baseline interoceptive awareness exhibited a negative descriptive correlation with perceived usefulness (*β* = −0.28, descriptive *p* = 0.087), thereby suggesting a hypothesis that students with lower baseline awareness may perceive greater value in external biofeedback.

eHealth literacy was positively associated with engagement (*β* = 0.30; descriptive *p* = 0.041). This association may reflect data-literacy skills, baseline motivation, or both.

Taken together, the acceptance-path results provide design hypotheses: ease of use, trust, and perceived usefulness may support engagement with abdominal-acoustic biofeedback. They should not be interpreted as causal evidence.

## 4 Discussion

The present exploratory randomized pilot study indicates that AbSound was feasible and acceptable over 4 weeks among non-clinical Taiwanese university students. The primary empirical contribution lies in short-term feasibility, engagement, user acceptance, and design requirements for abdominal-acoustic reflective prompting. The evidence should be interpreted at three levels: observed use and tolerability; exploratory self-reported behavioral signals relative to an active comparator; and preliminary *post hoc* event-detection agreement that still requires independent validation.

### 4.1 Interpretation of behavioral outcomes relative to an active comparator

Relative to active app-based dietary self-monitoring, AbSound users reported fewer late-night eating episodes, lower perceived eating-speed scores (interpreted as slower perceived eating), and higher brief dietary-quality scores over 4 weeks. The SREB difference was directionally favorable but marginal. These results are exploratory because outcomes were predominantly self-reported, the study was brief and modest in size, and the active comparator also received generic healthy-eating advice. The results may therefore reflect a combination of reflective prompting, novelty, expectancy, social desirability, and researcher contact rather than a definitive behavioral effect of abdominal-acoustic feedback.

The absolute behavioral changes may be practically relevant if replicated. Nevertheless, the extant data do not substantiate claims regarding the reversal of weight-gain trajectories, the reduction of caloric intake, the alteration of objectively measured meal pace, or the enhancement of clinical nutritional outcomes. No significant between-group differences in weight or waist circumference were observed, consistent with the brief follow-up period and the predominantly normal-BMI sample.

### 4.2 Psychological mechanisms and user experience

The findings of the Kano, TAM, and descriptive acceptance studies indicate that users engaged with AbSound when the device was comfortable, private, easy to use, and subjectively credible. This pattern is consistent with theories of HCI, self-monitoring, and engagement. However, the current design cannot isolate the active mechanism (23, 24, 45). It is hypothesized that the following mechanisms are plausible: interoceptive cueing, novelty, increased self-monitoring, perceived accountability, prompt salience, and demand characteristics.

Interpreting trust requires a high degree of caution. Users may place some trust in feedback they perceive as plausible, despite the absence of independent validation of the underlying physiological inference chain. Subsequent prototypes should communicate uncertainty transparently, allow users to control prompt frequency, and avoid deterministic language about hunger, satiety, overeating, or eating speed.

### 4.3 Physical tolerability and ecological limitations

Mild skin irritation during hot weather or perspiration, occasional false alerts in noisy restaurants, missed alerts during movement, and a potential increase in alert burden are not minor implementation details; they directly affect feasibility, trust, and adherence. In subsequent prototypes, it is necessary to enhance the materials that come into contact with the skin, facilitate easier repositioning, and incorporate options to pause and delete. Additionally, there is a requirement to characterize movement artifacts and environmental noise systematically, and to report false-positive, false-negative, signal-loss, and missingness rates under real-world conditions.

### 4.4 Clinical and ethical boundary

The present study did not include individuals with eating disorders, obesity requiring clinical treatment, gastrointestinal disease, or other medically vulnerable conditions. It therefore does not provide sufficient evidence to recommend AbSound as an adjunct to eating-disorder treatment, cognitive-behavioral therapy, obesity treatment, or clinical weight management. In users with restrictive eating, binge-spectrum symptoms, compulsive dietary control, or high body surveillance, repeated internal-signal prompts could heighten vigilance, guilt, confusion, or compulsive monitoring. Future studies in clinical or vulnerable populations would require screening, clinician involvement, explicit stopping rules, user-controlled notification settings, and monitoring for adverse psychological events (15–17, 30).

### 4.5 Study limitations

The study was brief, modest in size, and conducted at a single site. Its purpose was feasibility and development, not efficacy testing. The available pilot documentation does not allow retrospective reconstruction of sequence generation, responsibility for generating the sequence, assignment implementation, or allocation concealment; both allocation concealment and prospective registration therefore remain undocumented. These issues limit causal inference. Behavioral outcomes were predominantly self-reported; objective dietary intake, observed meal pace, appetite hormones, glucose, and repeated 24-h dietary recalls were not collected. The acoustic model was evaluated *post hoc* using selected reviewed segments rather than an independent physiological reference standard. TAM and contextual-variable analyses were limited to the intervention group, used adapted measures, and were underpowered for confirmatory modeling or moderation analysis. Finally, the cohort consisted of non-clinical Taiwanese university students with predominantly normal BMI; findings should not be generalized to clinical populations, eating-disorder risk groups, obesity treatment, gastrointestinal disease management, or long-term weight management.

### 4.6 Future directions

Future research must prioritize methodological validation before making any claims about broader implementation.

1. A longer, adequately powered trial should prespecify feasibility, behavioral, and nutritional endpoints; report sequence generation, allocation concealment, and trial registration; include intention-to-treat analysis; adjust or clearly prioritize multiple outcomes; and collect objective or repeated dietary reference measures, consistent with pilot-trial and complex-intervention guidance (27, 41, 46, 47).
2. Model development should report participant-independent train/test splits, class balance, blinded annotation, inter-rater reliability, class-specific sensitivity and specificity, positive predictive value, false-alert and missed-event rates, missingness, movement artifacts, signal loss, calibration, and ecological validation in noisy real-world settings (7, 8, 10).
3. Subsequent prototypes should combine uncertainty-aware feedback with user-controlled notification frequency, explicit false-alarm reporting, pause and delete options, and optional data-depth modes for users who want more detail without increasing the burden on others. These refinements should be described using replicable intervention-reporting frameworks and evaluated as design hypotheses, drawing on just-in-time adaptive intervention principles only after technical validation is stronger (39, 40, 45, 48, 49).
4. Integration with dietary guidance: With participants’ explicit consent, future studies could combine AbSound data with periodic dietary recalls or professional counseling. Such integration should be evaluated empirically rather than assumed to produce synergistic effects.
5. Studies in new populations: Extension beyond non-clinical Taiwanese university students should occur only after robust technical and behavioral validation. Studies involving medical vulnerability, eating-disorder risk, obesity treatment, or new cultural settings must include safety protocols, clinician involvement where appropriate, local privacy and usability assessment, and outcomes tailored to the target group.

## 5 Conclusion

This exploratory randomized pilot study demonstrates the short-term feasibility and acceptability of a user-centered abdominal-acoustic biofeedback system among non-clinical Taiwanese university students. Compared with an active dietary-tracking app comparator, AbSound was associated with exploratory self-reported reductions in late-night eating and perceived eating-speed scores, higher brief dietary-quality scores, and a marginally greater increase in SREB, with no clinically interpretable changes in weight or waist circumference.

The Kano-TAM-acceptance findings suggest that comfort, privacy, ease of use, subjective trust, and perceived usefulness are likely design priorities for future abdominal-acoustic dietary wearables. The acoustic model should be described as a preliminary event detector rather than a validated measure of hunger, satiety, dietary intake, diet quality, or objective eating speed. Before AbSound can be recommended for preventive care programs or vulnerable populations, larger, preregistered trials must include objective dietary assessment, participant-independent acoustic validation, longer follow-up, raw-data-based effect-size reporting, and explicit psychological-safety monitoring.

In summary, abdominal-acoustic biofeedback is an exploratory direction for low-burden dietary self-reflection, but the current evidence supports feasibility and hypothesis generation rather than translational readiness.

Future research should examine whether this approach remains acceptable over longer periods, whether behavioral changes persist beyond novelty effects, and whether benefits or risks vary by baseline interoceptive awareness, eHealth literacy, eating-related vulnerability, and cultural context.

## Data Availability

The aggregated data supporting the conclusions of this article are included in the article. Deidentified participant-level behavioral, wearable-sensor, and acoustic-event data are not publicly available because their sharing is constrained by participant privacy and the conditions of ethics approval. Requests for access may be directed to Tung Jing Fang (ftj@ntut.edu.tw) and will be considered for qualified researchers subject to institutional review and an appropriate data-use agreement.
